# A Cluster of Coronavirus Disease 2019 (COVID-19) Cases on an Inpatient Hospital Unit Involving Multiple Modes of Transmission

**DOI:** 10.1017/ash.2021.5

**Published:** 2021-07-29

**Authors:** Kelsey Witherspoon, Michael Haden, Justin Smyer, Jennifer Flaherty, Heather Smith, Nora Colburn, Christina Liscynesky, James Allen, Shandra Day

## Abstract

**Background:** The Ohio State University Wexner Medical Center identified a cluster of coronavirus disease 2019 (COVID-19) cases on an inpatient geriatric stroke care unit involving both patients and staff. The period of suspected severe acute respiratory coronavirus virus 2 (SARS-CoV-2) transmission and exposure on the unit was December 20, 2020, to January 1, 2021, with some patients and staff developing symptoms and testing positive within the 14 days thereafter. **Methods:** An epidemiologic investigation was conducted via chart review, staff interviews, and contact tracing to identify potential patient and staff linkages. All staff who worked on the unit were offered testing regardless of the presence of symptoms as well as all patients admitted during the outbreak period. **Results:** In total, 6 patients likely acquired COVID-19 in the hospital (HCA). An additional 6 patients admitted to the unit during the outbreak period subsequently tested positive but had other possible exposures outside the hospital (Fig. 1). One patient failed to undergo COVID-19 testing on admission but tested positive early in the cluster and is suspected to have contributed to patient to employee transmission. Moreover, 32 employees who worked on the unit in some capacity during this period tested positive, many of whom became symptomatic during their shifts. In addition, 18 employees elected for asymptomatic testing with 3 testing positive; these were included in the total. Some staff also identified potential community exposures. Additionally, staff reported an employee who was working while symptomatic with inconsistent mask use (index employee) early in the outbreak period. The index employee likely contributed to employee transmission but had no direct patient contact. Our epidemiologic investigation ultimately identified 12 employees felt to be linked to transmission based on significant, direct patient care provided to the patients within the outbreak period (Fig. 1). In addition, 3 employees had an exposure outside the hospital indicating likely community transmission. **Conclusions:** Transmission was felt to be multidirectional and included employee-to-employee, employee-to-patient, and patient-to-employee transmission in the setting of widespread community transmission. Interventions to stop transmission included widespread staff testing, staff auditing regarding temperature and symptom monitoring, and re-education on infection prevention practices. Particular focus was placed on appropriate PPE use including masking and eye protection, hand hygiene, and cleaning and disinfection practices throughout the unit. SARS-CoV-2 admission testing and limited visitation remain important strategies to minimize transmission in the hospital.

**Figure 1.**
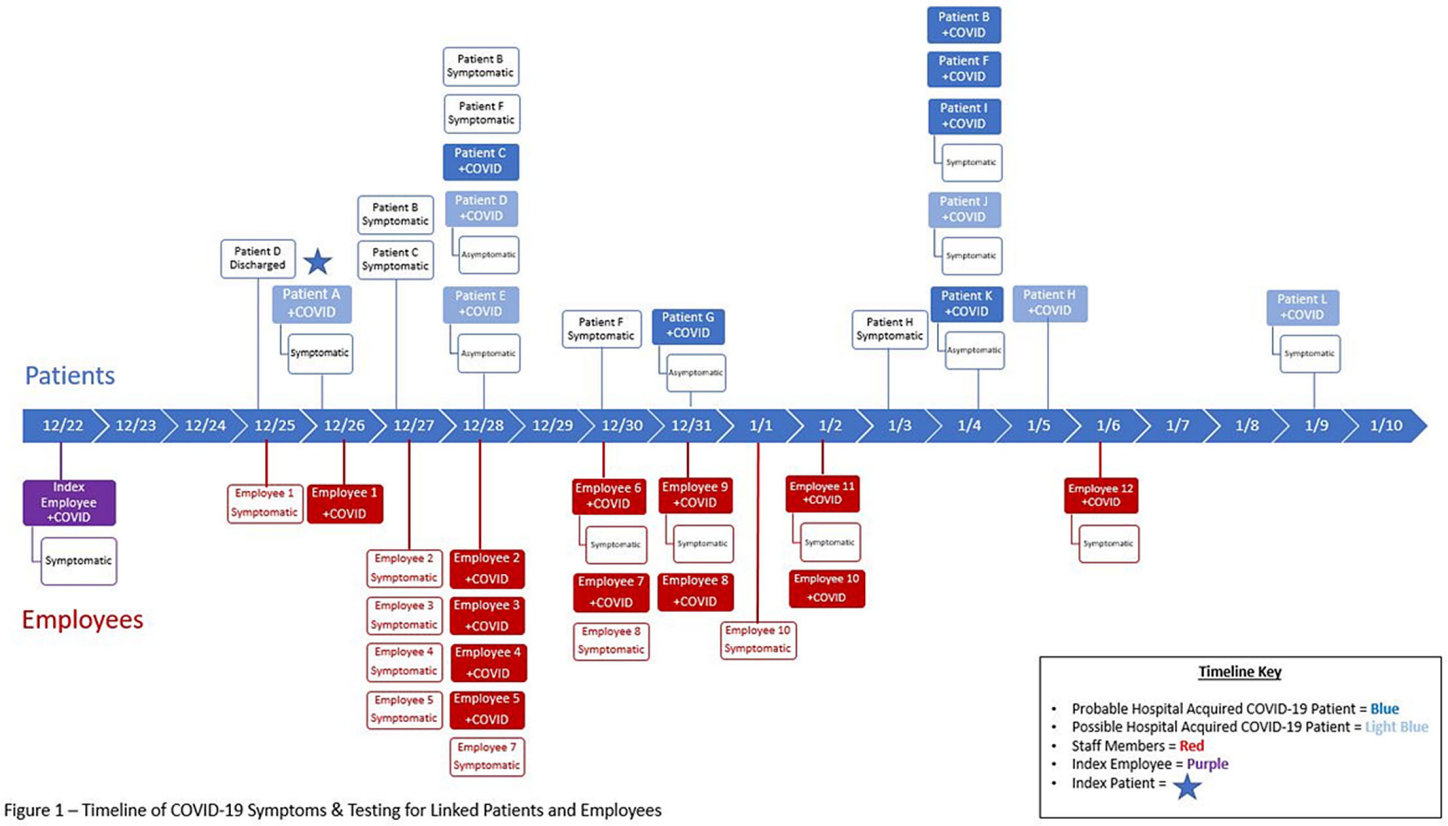

**Funding:** No

**Disclosures:** None

